# Delayed treatment initiation is associated with worse survival in screening-detected patients with upper gastrointestinal cancer in rural China: a 10-year cohort study

**DOI:** 10.7189/jogh.16.04293

**Published:** 2026-08-28

**Authors:** Ruyue Liu, Liangliang Wu, Chengxu Long, Chao Zheng, Xiaoyu Fang, Nan Zhang, Qiang Sun

**Affiliations:** 1 Department of Social Medicine and Health Management, School of Public Health, Cheeloo College of Medicine, Shandong University, Jinan, China; 2 NHC Key Lab of Health Economics and Policy Research (Shandong University), Jinan, China; 3 Center for Health Management and Policy Research, Shandong University (Shandong Provincial Key New Think Tank), Jinan, China; 4 Feicheng People's Hospital, Feicheng, China; 5 School of Medicine and Health Management, Huazhong University of Science and Technology, Wuhan, China; 6 Shandong Provincial Third Hospital, Cheeloo College of Medicine, Shandong University, Jinan, China; 7 Shandong Cancer Hospital and Institute, Shandong First Medical University and Shandong Academy of Medical Sciences, Jinan, China; 8 China National Health Development Research Center, Beijing, China

**Keywords:** upper gastrointestinal cancer, screening-positive patients, delayed TTI, survival impact, predictors

## Abstract

**Background:**

Time to treatment initiation (TTI) following cancer detection is a critical component of effective cancer control. In low-resource and rural settings, delays in TTI often reflect health system constraints and may undermine the survival benefits of screening programmes. However, evidence on the association between TTI and long-term survival among screening-positive patients with upper gastrointestinal cancer (UGC) in China is unknown.

**Methods:**

This retrospective cohort study included 265 screening-positive UGC patients identified from a population-based screening programme conducted between 2014 and 2015 in rural China (total screened population = 13,255). Time to treatment initiation was defined as the interval between confirmed diagnosis and initiation of treatment. Patients were categorised into ≤30 days, >30 days, and untreated groups. The primary outcome was 10-year overall survival (OS). Cox proportional hazards models were used to estimate hazard ratios (HRs), adjusting for demographic, socioeconomic, and clinical variables.

**Results:**

The median TTI was 55 days. Patients with TTI≤30 days had significantly higher 10-year OS (90.39%) compared with those with TTI>30 days and untreated patients. After adjustment, delayed treatment (>30 days) was associated with worse OS (HR = 2.50; 95% CI = 1.29–4.82) and cancer-specific survival (HR = 2.41; 95% CI = 1.21–4.79). Factors associated with delayed treatment included male, non-normal BMI, and earlier-stage lesions, indicating socioeconomic and structural barriers to timely treatment.

**Conclusions:**

Delays in treatment initiation represent a critical health system bottleneck that significantly compromises long-term survival among screening-detected UGC patients. A TTI threshold of 30 days may serve as a pragmatic quality-of-care indicator. Strengthening linkage-to-care pathways and addressing socioeconomic barriers are essential to maximise the effectiveness of cancer screening programmes in low-resource settings.

Cancer remains a leading cause of mortality worldwide, with a disproportionate burden in low- and middle-income countries (LMICs) [1]. Upper gastrointestinal cancer (UGC), including oesophageal and gastric cancers, remains a major cause of cancer-related mortality worldwide, with an estimated 1.48 million new cases and 1.10 million deaths in 2022 [1]. Nearly half of this burden occurs in China, where UGC ranks among the leading causes of cancer-related death [2,3]. In response, population-based screening programmes have been widely implemented in high-risk regions, improving early detection and increasing the proportion of cancers diagnosed at earlier, potentially curable stages.

While screening can shift diagnosis toward earlier stages, its effectiveness ultimately depends on timely access to treatment. Delays between diagnosis and treatment initiation, commonly measured as time to treatment initiation (TTI), are increasingly recognised as a critical determinant of cancer outcomes [4,5]. In resource-constrained settings, prolonged TTI often reflects health system limitations, including inadequate referral pathways, limited treatment capacity, and financial barriers [5,6]. Globally, delays in treatment initiation have been increasingly recognised as a major barrier to effective cancer control, particularly in LMICs where health system capacity is constrained. In addition to adverse clinical outcomes, delayed treatment may increase healthcare costs due to the need for more intensive therapies among patients [7]. Evidence from multiple malignancies, including breast [8], lung [9], colorectal [10], head and neck squamous cell carcinoma [11], cervical [4], muscle-invasive bladder [12], ovarian [13], and pancreatic cancers [14], has consistently shown that longer TTI is associated with worse survival. Evidence suggests that delays in treatment may attenuate or even negate the survival benefits of early detection. However, data on long-term outcomes associated with treatment delays in screening-detected populations, particularly in rural LMIC settings, remain limited.

A further challenge lies in the lack of consensus regarding the definition of delayed TTI. Previous studies have used highly variable thresholds, ranging from 7 to 90 days, often based on heterogeneous and sometimes arbitrary criteria [4,15–22]. Although a 30-day interval has been commonly adopted in studies of solid tumours [23,24] and is recommended by Chinese guidelines [25] for patients with positive screening results, this threshold has not been adequately validated in real-world screening settings. Existing work on determinants of delayed TTI has often focused on a single domain, such as sociodemographic characteristics or lifestyle factors, and has frequently relied on qualitative or cross-sectional designs [7,24,26–28].

In this context, we aimed to evaluate the impact of 30-day TTI cutoff on 10-year overall survival among screening-detected UGC patients in rural China and to identify socioeconomic and demographic factors associated with delayed treatment. We further sought to assess whether TTI could serve as a practical indicator of health system performance in cancer care delivery.

## METHODS

### Study design and participants

This retrospective cohort study included 13,255 residents aged 40–69 years who underwent endoscopic screening at Feicheng Hospital, a rural county-level hospital in Shandong Province, China, between 1 January 2014 and 31 December 2015. After screening, patients with high-grade intraepithelial neoplasia (HGIN) or carcinoma *in situ* (CIS) were identified based on pathological diagnosis (Table S1 in the **Online Supplementary Document**). All participants’ identifying information was handled in accordance with relevant ethical and legal requirements, and each eligible case was assigned a unique study code to ensure anonymity.

Screening-positive patients were included in the cohort if they met all prespecified eligibility criteria and had complete clinical and follow-up records. Clinical and survival data were cross-referenced with the Feicheng regional cancer registry and local all-cause mortality databases to enhance data accuracy and completeness. We excluded patients with major comorbidities (*i.e.* cardiovascular disease, chronic obstructive pulmonary disease, and severe renal impairment), those with advanced metastatic disease at diagnosis, and those with incomplete follow-up information. We presented characteristics of excluded patients and sensitivity analyses assessing the effect of these exclusions (Table S2 in the **Online Supplementary Document**).

The median follow-up duration was 120 months. Follow-up schedules were determined according to lesion type: patients with HGIN were followed once every 12 months, whereas those with CIS were followed once every six months. At each follow-up visit, changes in clinical status, treatment, and outcomes were systematically recorded using standardised forms. Treatment decisions were made entirely by patients and their treating physicians; the study investigators did not intervene in treatment choices or clinical recommendations.

### Outcomes and exposure

The primary outcome was overall survival (OS), defined as the time from diagnosis to death from any cause. The secondary outcome was cancer-specific survival (CSS), defined as the time from histopathological diagnosis to death due to UGC. Deaths attributable to UGC were coded as events, while patients alive at the end of follow-up or those who died from other causes were censored at the date of last contact or death.

The key exposure was TTI, defined as the interval (in days) between the date of histopathological diagnosis and the initiation of first definitive treatment, including endoscopic therapy and surgery. In this study, the histopathological diagnosis date was the date when the pathology report was issued. This date was obtained from the pathology report rather than the clinical records, which helps avoid inconsistencies across clinical documentation and ensures consistency in the time point used for TTI calculation.

### Variables and measurement

For survival analysis, the dependent variables were overall survival (OS) and cancer-specific survival (CSS). The key explanatory variable was TTI, categorised as ≤30 days, >30 days, and untreated. The untreated group was defined as patients who refused to receive treatment after histopathological diagnosis. Patients with incomplete clinical or follow-up information were excluded from the analysis to preserve the validity of outcome assessment.

For the analysis of factors associated with TTI, patient characteristics were used as predictors. These included demographic variables (age, gender, body mass index (BMI), and annual per capita household income) and clinical variables (family history of cancer and tumour stage (HGIN, CIS)).

### Statistical analysis

Differences in patient characteristics across the optimal TTI categories were assessed using the χ^2^ test or Fisher exact test, as appropriate. To evaluate the association between TTI and survival, we generated Kaplan-Meier plots and fitted multivariable Cox proportional hazards models. Multivariable models were adjusted for clinically relevant covariates, including age, gender, BMI, education status, number of family members, and tumour stage. Sensitivity analyses were performed using alternative TTI cutoffs (90 days and 180 days) to assess the robustness of the findings.

To further reduce potential confounding, propensity score-based inverse probability of treatment weighting (IPTW) was applied. Propensity scores were estimated using a logistic regression model including age, gender, BMI, education status, number of family members, and tumour stage. Weighted Cox proportional hazards models were then used to estimate the association between TTI and survival outcomes, with additional adjustment for tumour stage to ensure robustness.

Multivariable logistic regression models were used to identify significant predictors of delayed TTI. Differential associations were examined across predefined subgroups, including age (40–59 *vs.* 60–69 years), gender (male *vs.* female), BMI categories (normal (18.5–24.9kg/m^2^) *vs.* non-normal (<18.5kg/m^2^ or >24.9kg/m^2^)), and tumour stage (HGIN *vs.* CIS). Adjusted odds ratios (ORs), along with 95% CIs were generated from the multivariable regression models.

All statistical analyses were conducted using *R*, version 4.3.3 (R Foundation for Statistical Computing, Vienna, Austria), with statistical significance defined as a two-sided *P* < 0.05.

## RESULTS

### Population characteristics

**Figure 1:**
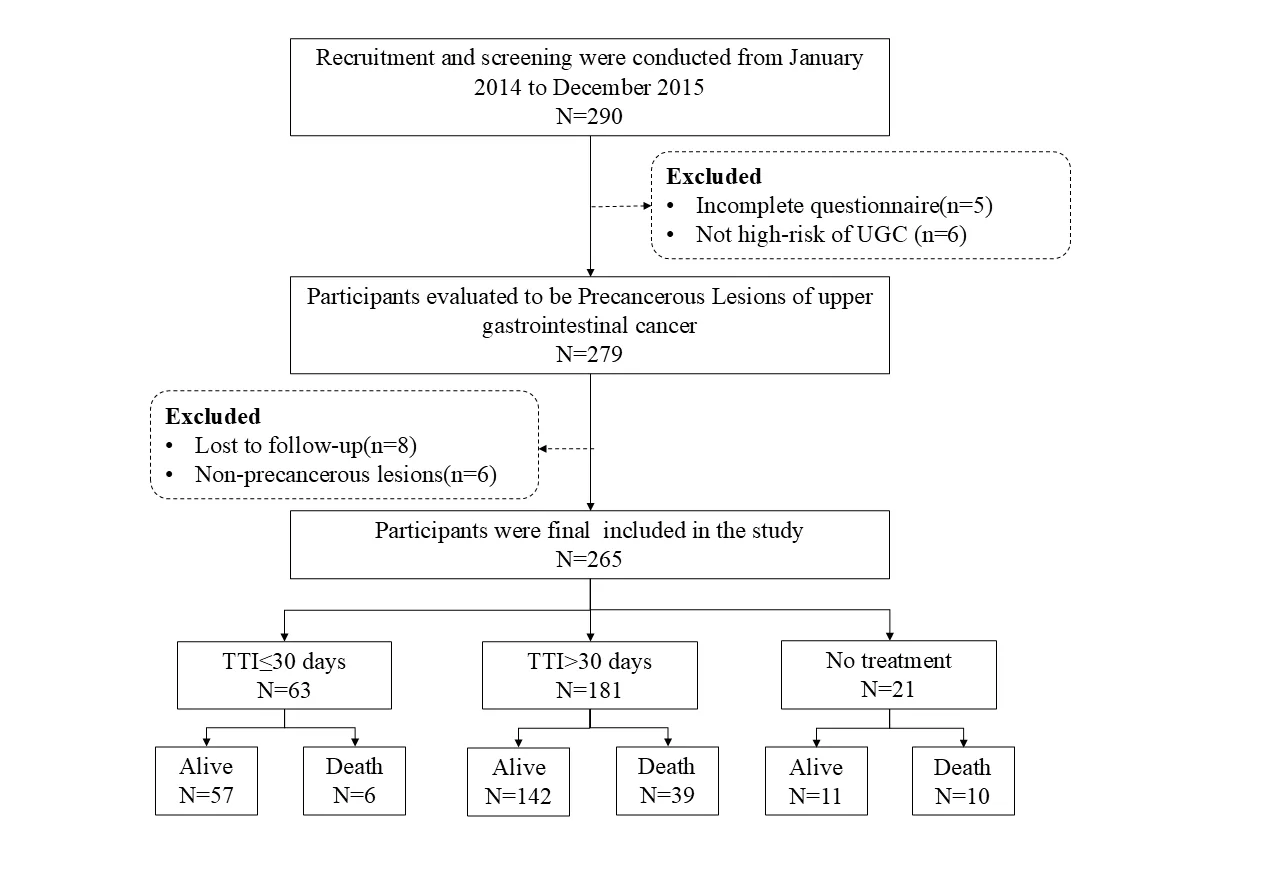
Study diagram and flowchart. HGIN – high-grade intraepithelial neoplasia, TTI – time to treatment initiation, UGC – upper gastrointestinal cancer.

After applying exclusion criteria, a total of 265 patients (mean ± SD age = 60.8 ± 18.2 years) were included, with a median follow-up of 120 months (**Figure 1**). In accordance with Chinese UGC guidelines, this study utilised 30 days as the threshold for analysis.

Patient characteristics are summarised (**Table 1**). Compared with patients with TTI of 30 days or less, a greater proportion of patients with TTI exceeding 30 days were 60–69 years of age (105 *vs.* 35) and male (137 *vs.* 41). Patients with TTI exceeding 30 days were also more likely to have a BMI of 18.5–24.9 kg/m^2^ (115 *vs.* 34), an annual per capita household income less than ¥50,000 (159 *vs.* 54). Among untreated patients, 71.43% were aged 60–69, 42.86% had a BMI of 18.5–24.9 kg/m^2^, and over half had an annual household income below ¥50,000. All χ^2^ statistics were not statistically significant (*P* > 0.05), indicating adequate balance across groups.

**Table 1:** Patient characteristics based on TTI threshold of 30 days

Variable	Total, No. (n = 265)	Patients by TTI, No. (%)			χ2 statistic
		**≤30 days, n = 63, n(%)**	**>30 days, n = 181, n (%)**	**Untreated n = 21, n (%)**	
Age, y					1.688
*40-59*	110	28 (44.44)	76 (41.99)	6 (28.57)	
*60-69*	155	35 (55.56)	105 (58.01)	15 (71.43)	
Gender					5.872
*Male*	197	41 (65.08)	137 (75.69)	19 (90.48)	
*Female*	68	22 (34.92)	44 (24.31)	2 (9.52)	
Marital status*					0.279
*Married*	243	58 (92.06)	169 (93.37)	20 (95.24)	
*Unmarried*	22	5 (7.94)	12 (6.63)	1 (4.76)	
Number of family members					2.294
*≤3*	127	33 (52.38)	87 (48.07)	7 (33.33)	
*>3*	138	30 (47.62)	94 (51.93)	14 (66.67)	
BMI (kg/m2)†					4.439
*Non-normal*	107	29 (46.03)	66 (36.46)	12 (57.14)	
*Normal*	158	34 (53.97)	115 (63.54)	9 (42.86)	
Annual per capita household income (CNY)					1.349
*≥50,000*	32	9 (14.29)	22 (12.15)	1 (4.76)	
*<50,000*	233	54 (85.71)	159 (87.85)	20 (95.24)	
Education status					3.384
*Up to primary school*	31	5 (7.94)	25 (13.81)	1 (4.76)	
*Middle school and above*	205	49 (77.78)	138 (76.24)	18 (85.71)	
*Unknown*	29	9 (14.29)	18 (9.95)	2 (9.53)	
Family history of cancer					2.355
*No*	214	53 (84.13)	142 (78.45)	19 (90.48)	
*Yes*	51	10 (15.87)	39 (21.55)	2 (9.52)	
Stage					5.519
*HGIN*	112	16 (25.40)	72 (39.78)	5 (23.81)	
*CIS*	153	47 (74.60)	109 (60.22)	16 (76.19)	

BMI – body mass index, UGC – upper gastrointestinal cancer, HGIN – high-grade intraepithelial neoplasia, CIS – carcinoma *in situ*, TTI – time to treatment initiation

*Marital status: classified as married or unmarried (including divorced, widowed, never married).

†BMI normal was defined as 18.5 – 24.9 kg/m^2^, whereas non-normal BMI was defined as <18.5 kg/m^2^ or >24.9 kg/m^2^.

### Association of TTI with survival outcomes

The 10-year overall survival was 79.49% (95% CI = 74.8-84.6) for all patients, 51.58% (95% CI = 33.88–78.53%) for patients with no treatment, 78.91% (95% CI = 73.17–85.10%) for patients with TTI exceeding 30 days, and 90.39% (95% CI = 83.37–98.01%) for patients with TTI of 30 days or less (Table S3 in the **Online Supplementary Document**).

**Figure 2:**
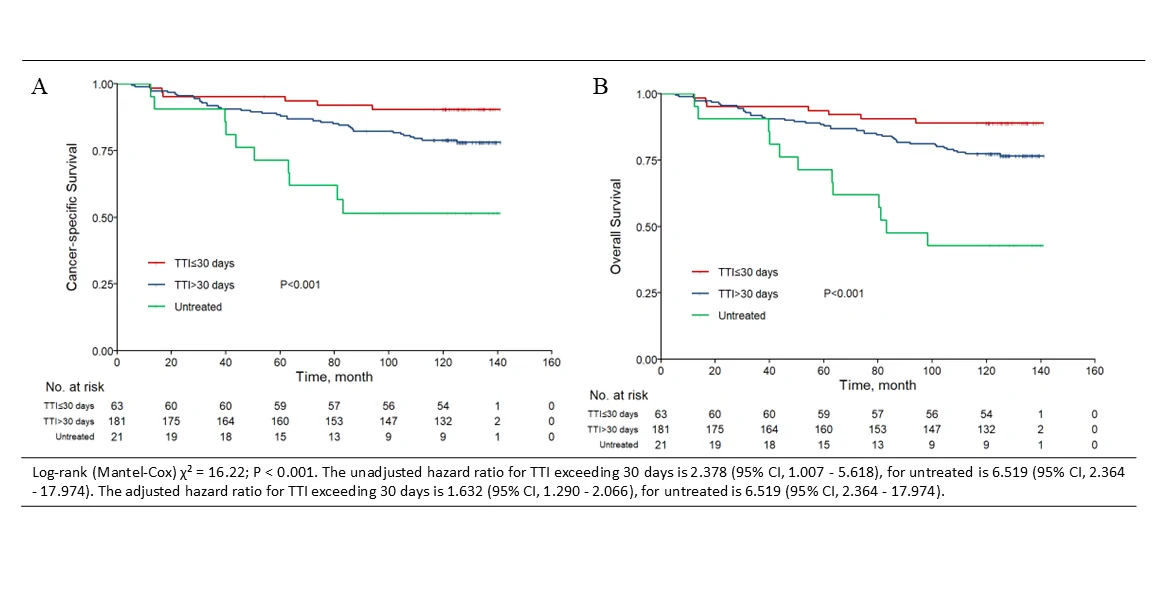
Survival outcomes according to time to treatment initiation after diagnosis. Panel A. Cancer-specific survival (CSS). Panel B. Overall survival (OS). Kaplan-Meier curves stratified by time to treatment initiation (TTI≤30 days, TTI>30 days, and untreated). Survival differences were compared using the log-rank test (*P* < 0.001). Numbers at risk are presented below the x-axis. CI – confidence interval, TTI – time to treatment initiation.

Kaplan-Meier survival analysis showed clear differences in survival among patients who were untreated, had a TTI longer than 30 days, or had a TTI of 30 days or less (**Figure 2**, Panels A–B), with the curves beginning to separate between approximately 40 and 70 months (Log-rank, χ^2^ = 16.22, *P* < 0.001). Consistent patterns were observed for CSS, with delayed or absent treatment associated with a substantially higher risk of cancer-related mortality. To evaluate the robustness of these findings, sensitivity analyses were performed using alternative TTI thresholds of 90 and 180 days (Figure S1 in the **Online Supplementary Document**). Across both cutoffs, delayed treatment remained consistently associated with poorer OS and CSS.

In the unweighted Cox regression analysis (Table S4 in the **Online Supplementary Document**), older age and non-normal BMI were significantly associated with worse CSS and OS. Patients with BMI of 18.5–24.9 kg/m^2^ had substantially lower mortality risks compared with those with BMI<18.5 kg/m^2^ or >24.9 kg/m^2^. TTI>30 days was also associated with significantly increased risks of both OS (HR = 2.96; 95% CI = 1.49–5.90) and CSS (HR = 2.87; 95% CI = 1.38–5.94).

After IPTW adjustment (**Table 2**), these associations remained largely consistent. Older age remained independently associated with poorer OS (HR = 1.08; 95% CI = 1.03–1.14) and CSS (HR = 1.08; 95% CI = 1.02–1.13). Normal BMI was associated with reduced mortality risk (HR = 0.44; 95% CI = 0.27–0.74). In addition, TTI>30 days remained significantly associated with worse outcomes, with increased risks of OS (HR = 2.50; 95% CI = 1.29–4.81) and CSS (HR = 2.41; 95% CI = 1.21–4.79).

**Table2:** Inverse probability of treatment weighting-adjusted Cox regression analysis for overall survival and cancer-specific survival, stratified by stage*

Variables	Overall survival		Cancer-specific survival	
	**HR (95% CI)**	***P*-value**	**HR (95% CI)**	***P*-value**
Age	1.08 (1.03–1.14)	0.002	1.08 (1.02–1.13)	0.006
Gender				
*Male*	Reference		Reference	
*Female*	0.62 (0.33–1.19)	0.150	0.54 (0.27–1.11)	0.093
BMI (kg/m^2^)				
*Non-normal (<18.5 kg/m^2^ or >24.9 kg/m^2^)*	Reference		Reference	
*Normal (18.5–24.9 kg/m^2^)*	0.39 (0.24–0.64)	<0.001	0.44 (0.27–0.74)	0.002
Number of family members				
*≤3*	Reference		Reference	
*>3*	1.98 (1.17–3.35)	0.011	2.01 (1.15–3.53)	0.014
Education status				
*Up to primary school*	Reference		Reference	
*Middle school and above*	0.56 (0.28–1.15)	0.114	0.51(0.24–1.06)	0.070
*Unknown*	0.15(0.02–0.45)	0.002	0.11 (0.02–0.47)	0.003
TTI				
*<30 days*	Reference		Reference	
*>30 days*	2.50 (1.29–4.81)	0.006	2.41(1.21–4.79)	0.012

OS – overall survival, CSS – cancer-specific survival, HR – hazard ratio, TTI – time to treatment initiation

*Weighted stratified Cox model (stage-stratified) with TTI as the main predictor for CSS, showing concordance C = 0.745; likelihood ratio test = 45.16 (*P* < 0.01), Wald test = 49.72 (*P* < 0.01), and log-rank (score) test = 42.4 (*P* < 0.01); robust = 36.41 (*P* < 0.01). Weighted stratified Cox model (stage-stratified) with TTI as the main predictor for OS, showing concordance C = 0.755; likelihood ratio test = 53.58 (*P* < 0.01), Wald test = 58.17 (*P* < 0.01), and log-rank (score) test = 49.94 (*P* < 0.01); robust = 42.54 (*P* < 0.01). For CSS, the E-value was 4.28 (based on the hazard ratio) and 1.72 (based on the lower bound of the 95% CI). For OS, the E-value was 4.56 and 1.78, respectively.

### Factors related to delayed TTI

The binary logistic regression was conducted to explore the factors associated with delayed TTI exceeding 30 days (**Table 3**). Compared with individuals aged 40–59 years, those aged 60–69 years had higher odds of delayed TTI (OR = 1.07; 95% CI = 0.57–1.99), although the association was not statistically significant. Females had a lower likelihood of delayed TTI than males, with an OR of 0.39 (95% CI = 0.20–0.78). For financial factors, an annual per capita income of less than ¥50,000 correlated with increased odds of delayed TTI (OR = 0.77; 95% CI = 0.31–1.88). Patients with a BMI of 18.5 to 24.9 had an OR of 0.32 (95% CI = 0.17–0.59) compared to those with a non-normal BMI. Having a family history of cancer was linked to an OR of 1.70 (95% CI = 0.76–3.81). Compared with patients with HGIN, those CIS were less likely to experience delayed TTI (OR = 0.39; 95% CI = 0.20–0.75). Overall, the model showed a significantly better fit than the null model (Likelihood Ratio Test = 258.98, *P* < 0.001), indicating that the included covariates are collectively associated with the outcome.

**Table 3:** Multivariable logistic regression model to examine factors influencing delayed time to treatment initiation

Variable	TTI of 30 days or less	TTI exceeding 30 days*
	**OR (95%CI)**	***P-*value**
Age of 60–69 years *vs. *40–59 years	1.07 (0.57–1.99)	0.832
Female *vs.* male	0.39 (0.20–0.78)	0.007
Annual per capita household income ≥50,000 CNY *vs.*<50,000 CNY	0.77 (0.31–1.88)	0.546
BMI normal *vs* non-normal†	0.32 (0.17–0.59)	<0.001
Have family history of cancer *vs.* do not have	1.70 (0.76–3.81)	0.195
Tumour stage of CIS *vs.* HIGN	0.39 (0.20–0.75)	0.005
Model: Chisq 2 = 258.98, *P* < 0.001		

BMI – body mass index, OR – odds ratio, TTI – time to treatment initiation

*TTI exceeding 30 days including cases of TTI exceeding 30 days and cases with no treatment.

†BMI normal was defined as 18.5 – 24.9 kg/m^2^, whereas non-normal BMI was defined as <18.5 kg/m^2^ or >24.9 kg/m^2^.

## DISCUSSION

This study evaluated the impact of TTI on long-term survival among screening-detected patients with UGC, and identified key factors associated with treatment delay. Our findings demonstrated that TTI exceeding 30 days was significantly associated with worse survival, highlighting the critical importance of timely treatment following cancer screening.

Delayed TTI was associated with poorer survival outcomes in screening-detected UGC patients. Several mechanisms may explain this relationship. First, delays in treatment may allow for tumour progression and potential stage migration, thereby reducing the likelihood of curative intervention. Second, screening-detected cancers are typically identified at earlier stages, representing a critical window during which timely treatment can achieve optimal outcomes. Delays beyond this window may therefore disproportionately compromise the survival benefits conferred by early detection. Unlike symptomatic cancers, delays in screening-detected cases may represent missed opportunities for curative treatment.

Consistent with our findings, prior studies across multiple malignancies have demonstrated that prolonged TTI is associated with worse survival outcomes [4,8,11,12]. A recent analysis [8] of breast cancer using the SEER-Medicare and NCDB databases revealed that the time from diagnosis to surgery significantly affects survival rates; specifically, each additional time interval increased the hazard of mortality. Another observational study also highlighted a significant negative correlation between time to initial treatment and survival across various cancer types [14]. Research by Gore JL *et al.* [22] showed that patients undergoing radical cystectomy with delays exceeding 12 weeks encountered a significant increase in mortality risk. However, most of these studies focused on symptomatic populations. Evidence regarding screening-detected cancers remains limited. Our study extends the existing literature by specifically examining this association in a screening context, where timely treatment is essential to fully realise the benefits of early detection programmes.

The cut-off of TTI threshold still lacks a unified standard, with various studies employing cut-offs ranging from 7 to 90 days across different cancer types [4,16–22]. The most commonly used threshold is 30 days [23]. Setting a single cutoff to define treatment delay is useful for establishing a benchmark for quality of care and for determining what constitutes a tolerable amount of delay. However, to our knowledge, no study has specifically explored the optimal TTI threshold for UGC in China. According to the guidelines for UGC, a TTI of 30 days or less is deemed optimal [25].

Based on our calculated threshold of 30 days, 76.23% patients experienced treatment delay. This is higher than previous study conducted in China, which demonstrated that 36% of patients with UGC experience treatment delays [29]. Liao DZ *et al.* [11] reported that 20.8% patients with head and neck squamous cell carcinoma experienced delays exceeding 60 days, which was associated with poorer overall survival. In another study defining a treatment delay as three months, the delay rate was 58.8% [15]. This variation in treatment delays may be related to differing cut-off settings, as well as variations in countries and populations. These findings highlight the importance of interpreting treatment delay within specific healthcare contexts. Therefore, we should focus on the Chinese population and specific cut-offs to accurately report the status of treatment delays, providing valuable reference points for policymakers to formulate appropriate laws and policies.

There has been less research on the predictors of and reasons for treatment delay in upper gastrointestinal cancer, especially among screening-positive patients. We found that male, earlier-stage lesions, and a non-normal BMI (below 18.5 or up 24.9kg/m^2^) are associated with a higher likelihood of experiencing delays in treatment. These factors likely reflect a combination of socioeconomic barriers, healthcare accessibility, and patient-level health status. In contrast to other studies, males had significantly lower proportion of delayed TTI [23]. A cross-sectional study in Malaysia found that factors influencing presentation delay among cancer patients include socioeconomic status, emotional barriers and anxiety about follow-up investigations, and long waiting times [24]. An earlier study of colorectal cancer found that abdominal pain and/or vomiting and advice from family members or associates prompted earlier medical consultation, whereas localised rectal symptoms and weight loss were associated with longer delays [26]. Patients who were examined by their doctors were also referred for surgical assessment sooner [26]. Some studies identify that treatment delays and prolonged hospital stay in inpatient cancer care are associated with patient characteristics (such as obesity, older age, and comorbidities), irregular medical processes, the need for additional tests, and lack of health insurance [30], leading to increased costs and poorer patient outcomes. In addition, patients with earlier-stage disease may perceive their condition as less urgent, whereas clinicians may prioritise more advanced cases, potentially influencing treatment timing. Identifying specific influencing factors will provide a reference for our next steps in implementing targeted interventions for patients, ultimately enhancing their survival rates.

Our findings suggest several practical and research implications. Clinically, the high proportion of delayed TTI highlights the need to streamline referral and treatment pathways after positive UGC screening, especially for high-risk groups (older adults, men, those with lower income, larger families). Targeted patient navigation, health education on timely treatment, and social or financial support may help reduce delays. From a public health perspective, improving timeliness of treatment may enhance the overall effectiveness of cancer screening programmes. For research, these results underscore the importance of using context-specific TTI cut-offs in Chinese populations when assessing quality of care and survival. Future multi-centre prospective studies should validate the 30-day threshold, and evaluate interventions (*e.g.* navigation programmes, reminder systems, policy reforms) aimed at shortening TTI and improving survival in screening-positive UGC patients.

Several limitations of this study existed. First, as a retrospective study, it is subject to potential inaccuracies in the cancer registry and patient medical records, including miscoding. Second, this was a single-institution study with a relatively small sample size. Future studies should be conducted in larger, multi-centre cohorts to validate our findings. Third, there were missing data. Multiple imputation was not performed because the missingness mechanism could not be reasonably assumed to be missing at random, and inappropriate imputation might have introduced additional bias and uncertainty. However, sensitivity analyses indicated that excluding this subset of patients did not materially change the results. Fourth, although we adjusted for measured covariates and applied eligibility criteria to limit certain confounding, residual confounding cannot be fully ruled out in observational studies, including unmeasured factors such as clinician- or institution-level characteristics that may influence TTI, as well as patient physical health that could introduce indication bias. In future studies, we will further focus on these factors and explore their potential roles in shaping TTI and patient outcomes. Finally, although the main models were adjusted for measured confounders and subgroup analyses were pre-specified by stratification variables, we did not include TTI–stratum interaction terms in the main model. This was because limited sample sizes within subgroups may yield unstable interaction estimates. Nevertheless, we used IPTW to better balance measured baseline covariates across TTI groups, thereby reducing confounding when estimating stratified TTI effects.

## CONCLUSIONS

Health system delays in treatment initiation significantly compromise survival among screening-detected UGC patients. Addressing these delays through improved care pathways, resource allocation, and reduction of socioeconomic barriers is essential to maximise the effectiveness of cancer screening programmes in low-resource settings.

## Additional material


Online Supplementary Document

## Data Availability

**Data availability:** The data analysed in this study are available from the corresponding author upon reasonable request.
