# Supplementary material for: Delayed treatment initiation is associated with worse survival in screening-detected patients with upper gastrointestinal cancer in rural China: a 10-year cohort study
Source: J Glob Health. 2026 Aug 28;16:04293. doi: 10.7189/jogh.16.04293 (PMC13522900; doi:10.7189/jogh.16.04293)
Supplement: Supplementary file 1 [file jogh-16-04293-s001.pdf]

Supplement to: Liu R, Wu L, Long C, Zheng Chao, Fang X, Zhang N, Sun Q. Delayed Treatment Initiation Is Associated with Worse Survival in Screening-detected patients with Upper Gastrointestinal Cancer in Rural China: a 10-year cohort study. J Glob Health. 2026; 16:04293.

Table of contents

Table S1. Pathology Codes and Result Interpretation Criteria.

Table S2. Sensitivity analysis of the excluded and included populations.

Table S3. Overall Survival Rates.

Figure S1. Kaplan–Meier survival curves for CSS and OS according to different TTI cutoffs (90 days and 180 days).

Table S4. Unweighted Cox regression analysis of overall survival (OS) and cancer-specific survival (CSS).

Table S1. Pathology Codes and Result Interpretation Criteria.

Code	Diagnosis	Result	Stage	Code	Diagnosis	Result	Stage
1	Normal squamous epithelium	Negative	—	186	Barrett esophagus	Negative	—
4	Basal cell hyperplasia	Negative	—	19	Indefinite for intraepithelial neoplasia in glandular epithelium	Negative	—
5	Mild esophagitis	Negative	—	20	Low-grade intraepithelial neoplasia of glandular epithelium	Negative	—
6	Moderate esophagitis	Negative	—	21	High-grade intraepithelial neoplasia of glandular epithelium	Positive	Early
7	Severe esophagitis	Negative	—	22	Intramucosal adenocarcinoma (not otherwise specified)	Positive	Early
8	Low-grade squamous intraepithelial neoplasia	Negative	—	221	Intramucosal adenocarcinoma	Positive	Early
10	High-grade squamous intraepithelial neoplasia	Positive	Early	222	Submucosal adenocarcinoma	Positive	Early
11	Squamous intraepithelial neoplasia, not otherwise specified (NOS)	Negative	—	23	Adenocarcinoma	Positive	Advanced
13	High-grade squamous intraepithelial neoplasia, suspicious for invasion	Positive	Early	24	Adenosquamous carcinoma	Positive	Advanced
14	Intramucosal squamous cell carcinoma, not otherwise specified	Positive	Early	25	Neuroendocrine carcinoma (NEC, including small cell carcinoma and large cell neuroendocrine carcinoma)	Positive	Advanced
141	Intramucosal squamous cell carcinoma	Positive	Early	26	Neuroendocrine tumor (NET G1, G2, G3)	Positive	Advanced
142	Submucosal squamous cell carcinoma	Positive	Early	27	Carcinoma, not otherwise specified (NOS)	Positive	Advanced
15	Squamous cell carcinoma	Positive	Advanced	28	Lymphoma	Positive	Advanced
16	Normal glandular epithelium	Negative	—	29	Mesenchymal tumor	Positive	Advanced
17	Non-atrophic gastritis	Negative	—	30	Squamous papilloma of the esophagus	Negative	—
18	Atrophic gastritis/intestinal metaplasia, not otherwise specified	Negative	—	31	Fundic gland polyp	Negative	—
181	Mild–moderate atrophic gastritis	Negative	—	32	Hyperplastic polyp	Negative	—
182	Severe atrophic gastritis	Negative	—	33	Others	Negative	—
183	Mild–moderate intestinal metaplasia	Negative	—	99	Insufficient for diagnosis	Negative	—
184	Severe intestinal metaplasia	Negative	—	100	No biopsy required and no pathological diagnosis required	Negative	—

Endoscopic diagnosis is for reference only; the final diagnosis is determined by histopathological examination of biopsy specimens. Only high-grade lesions and early-stage cancers were included in this study.

Table S2. Sensitivity analysis of the excluded and included populations.

Variable	Included patients (N=265)	Excluded Patients (N=25)	χ^2 Statistic	p-value
Age, y			0.022	0.884
40-59	110	10		
60-69	155	15		
Gender			1.252	0.263
Male	197	16		
Female	68	9		
Marital status			1.659	0.198
Married	243	21		
Unmarried	22	4		
Number of family members			0.141	0.707
⩽3	127	11		
> 3	138	14		
BMI (kg/m^2)*			2.576	0.108
Non-Normal	107	6		
Normal	158	19		
Annual per household income†			1.289	0.256
≥50000 CNY	32	5		
<50000 CNY	233	20		
Education Status			2.317	0.314
Up to primary school	31	5		
Middle school and above	205	16		
Unknown	29	4		
Family history of cancer			2.293	0.130
No	214	17		
Yes	51	8		
Stage			0.307	0.579
HGIN	112	12		
CIS	153	13		

Marital status: Classified as married or unmarried (including divorced, widowed, never married).
BMI-Body Mass Index; UGC-Upper gastrointestinal cancer.

* BMI normal was defined as 18.5-24.9 kg/m^2, whereas non-normal BMI was defined as <18.5 kg/m^2 or >24.9 kg/m^2.

†Annual per household income: Calculated as the average annual income per household member.

Table S3. Overall Survival Rates.

Groups	Survival rate (10-year), %	95%CI
Overall	79.49	74.76, 84.53
TTI ≤30 days	90.39	83.37, 98.01
TTI > 30 days	78.91	73.17, 85.10
Untreated	51.59	33.88, 78.53

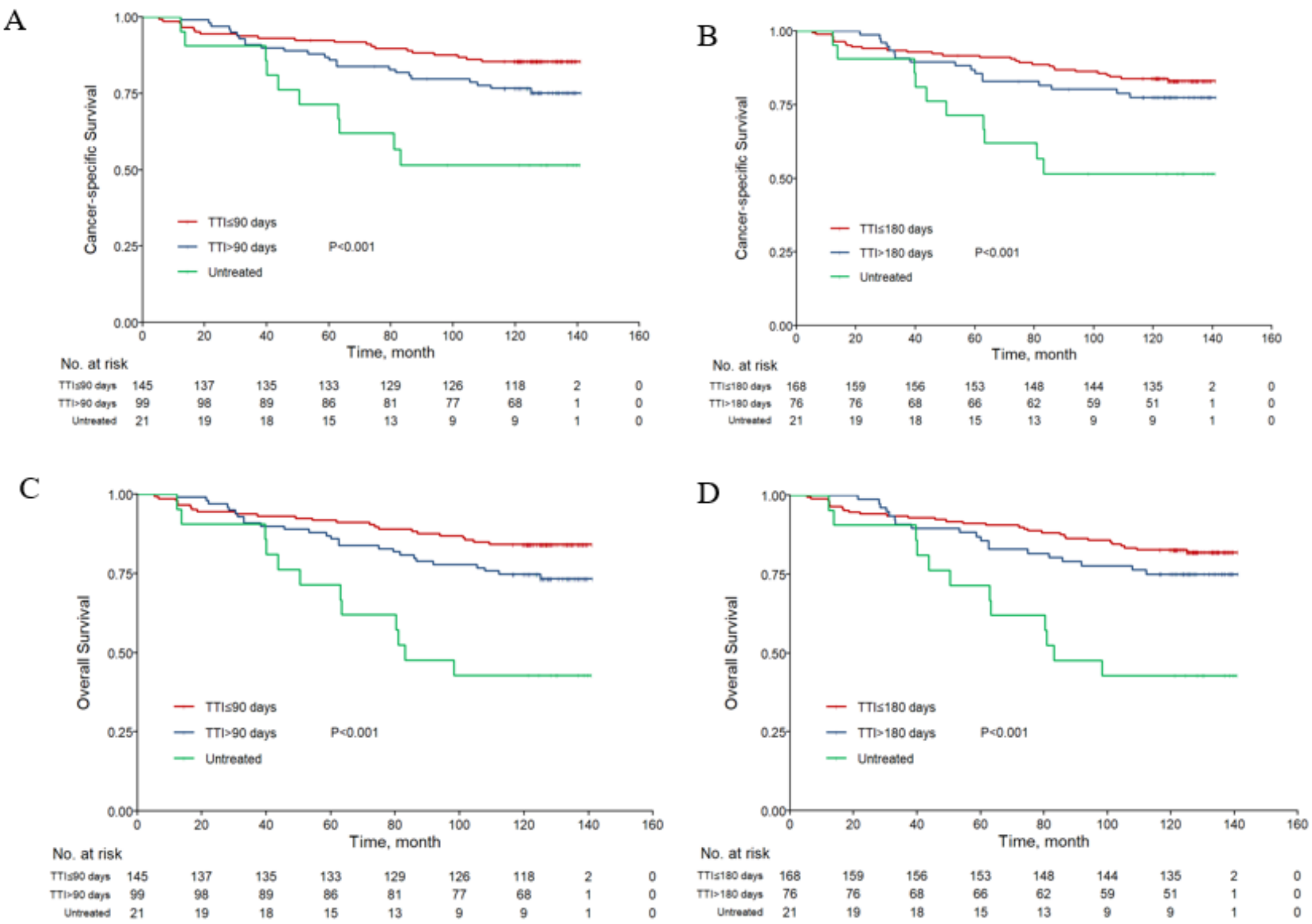

Figure S1. Kaplan–Meier survival curves for CSS and OS according to different TTI cutoffs (90 days and 180 days). (A) CSS stratified by TTI using a 90-day cutoff; (B) CSS stratified by TTI using a 180-day cutoff; (C) OS stratified by TTI using a 90-day cutoff; (D) OS stratified by TTI using a 180-day cutoff. Patients were categorized into TTI ≤ cutoff, TTI > cutoff, and untreated groups. Earlier treatment initiation was associated with significantly improved survival outcomes compared with delayed or no treatment (all $P < 0.001$).

TTI-time to treatment initiation.

Table S4. Unweighted Cox regression analysis of cancer-specific survival (CSS) and overall survival (OS)

Variables	Overall Survival, OS		Cancer-specific survival, CSS	
	HR (95%CI)	p-value	HR (95%CI)	p-value
Age	1.07(1.02-1.13)	0.005	1.08 (1.02-1.13)	0.011
Gender				
Male	Reference		Reference	
Female	0.66 (0.34-1.28)	0.218	0.59 (0.29-1.21)	0.152
BMI (kg/m^2)				
<18.5	Reference		Reference	
18.5-24.9	0.40 (0.24-0.67)	<0.001	0.46 (0.24-0.71)	0.004
Number of family members				
Non-Normal (<18.5 kg/m^2 or >24.9 kg/m^2)	Reference		Reference	
Normal (18.5-24.9 kg/m^2)	1.99(1.14-3.48)	0.015	1.99 (1.11-3.58)	0.020
Education Status				
Up to primary school	Reference		Reference	
Middle school and above	0.65 (0.31-1.36)	0.253	0.59 (0.28-1.28)	0.186
Unknown	0.12 (0.03-0.59)	0.009	0.13 (0.03-0.63)	0.011
TTI				
<30 days	Reference		Reference	
>30 days	2.96(1.49-5.90)	0.002	2.87(1.38-5.94)	0.004

CSS-cancer-specific survival; OS-Overall Survival; BMI- Body Mass Index; HR- hazard ratio; TTI-time to treatment initiation.
